# Associations between ultraviolet radiation, tree cover and adolescent sunburns

**DOI:** 10.1186/s12942-020-00253-x

**Published:** 2020-12-14

**Authors:** Calvin P. Tribby, Anne K. Julian, April Y. Oh, Frank M. Perna, David Berrigan

**Affiliations:** 1 Department of Geography, University of Hong Kong, Room 1023, 10th Floor, The Jockey Club Tower Centennial Campus, Pokfulam Road, Hong Kong, Hong Kong; 2 Health Behaviors Research Branch, Behavioral Research Program, Division of Cancer Control and Population Sciences, National Cancer Institute, Bethesda, MD USA; 3 Cancer Prevention Fellowship Program, Division of Cancer Prevention, National Cancer Institute, Bethesda, MD USA; 4 Implementation Science Team, Office of the Director, Division of Cancer Control and Population Sciences, National Cancer Institute, Bethesda, MD USA

**Keywords:** Sunburn, Ultraviolet radiation, Tree cover, Shade, Adolescents, School

## Abstract

**Background:**

Sunburn is the strongest risk factor for melanoma and non-melanoma skin cancers. Adolescent sunburns are related to higher risk of developing melanoma later in life. Little is known about the association of sunburns and shade, particularly tree cover, around adolescent homes and schools. This linkage study assessed associations of adolescent self-reported sunburns with ambient ultraviolet radiation (UV) and tree cover.

**Methods:**

We analyzed a U.S. national sample of parent–child dyads (n = 1333) from the 2014 Family Life, Activity, Sun, Health, and Eating (FLASHE) study conducted by the National Cancer Institute. The outcome was adolescent sunburns reported for the previous 12 months. GIS buffers around geocoded home and school addresses were used to summarize UV and tree cover. A sensitivity analysis assessed different UV measures and tree cover buffer distances. Logistic regression models estimated the adjusted odds of any sunburns for five models: (1) no environmental variables; (2) spatial variables of latitude and longitude; (3) UV; (4) tree cover; and, (5) a combined model with UV and tree cover. Covariates included common sunburn-related items such as sun protective behaviors, socio-demographics, and latitude. Model residuals were assessed for spatial dependency and clustering.

**Results:**

Overall, 44% of adolescents reported any sunburns in the previous 12 months. For the bivariate associations, lower categories of UV were associated with any reported sunburns (*p-trend* = 0.002). Home tree cover was not associated with any reported sunburns (*p-trend* = 0.08), whereas schools with lower categories of tree cover were associated with sunburns (*p-trend* = 0.008). The adjusted odds of any sunburns by UV tertiles, as a linear tread, was 0.89 (0.76–1.05) (*p* = 0.17); school tree cover was: 0.91 (0.78–1.07) (*p* = 0.25). Neither UV nor tree cover, in a combined model, were significant. Sensitivity analyses resulted in the optimal buffer size of 200 m for summarizing tree cover. Spatial dependence of residuals was not significant and clustering was significant for about 6% or less of the sample in each model.

**Conclusions:**

We did not find significant relationships between UV or tree cover and adolescent sunburns, when adjusted by sunburn-related covariates. Better contextual data about where sunburns occurred is needed to identify environmental correlates of sunburn.

## Background

Reducing exposure to ultraviolet radiation (UV) across the life course could reduce the risk of melanoma and non-melanoma skin cancers [1–3]. Sunburn, caused by over-exposure to UV, is the most clinically-relevant risk factor for melanoma, with those experiencing more sunburns having higher risk of melanoma [4]. Adolescence is an important life stage to study sunburns, as sunburns during this age are related to higher risk of developing melanoma later in life [4] and the majority of lifetime UV exposure occurs before the age of twenty [5].

Globally, the possible role of shade in reducing UV exposure and potentially sunburns is not well understood. Shade is one attribute of the environment that may reduce daily UV exposure [6–8]. Previous research has found that in addition to ambient UV, other environmental characteristics such as lower latitude, less daily rainfall and summer season were predictors of increased daily personal UV exposure [9–11]. Similar correlations have not yet been assessed for environmental shade and sunburns.

Health behaviors related to sun exposure are also associated with sunburns. For example, individuals with higher levels of physical activity in Australia and the U.S. are associated with increased odds of sunburns [12–14]. These associations are likely because physical activity often occurs outdoors. However, few studies have measured time spent outdoors, physical activity and sunburn risk concurrently. Walking, the most common physical activity in the U.S., was not generally associated with sunburns [15]. One sun protective behavior, using sunscreen, is associated with increased odds of sunburns [13], whereas other sun protective behaviors, such as seeking shade, wearing a hat, and sun protective clothing, are associated with decreased risk of sunburns [16]. Knowledge of how physical activity and sun protective behaviors interact to influence sunburn risk is an evolving and active research area [17].

The prevalence of adolescent sunburns, defined as one or more sunburn in the past summer, is consistently high in developed countries, for example: in the U.S., 72% [18], in Denmark, 61% [19] and in Australia, 66% [20]. Prospectively, as children age into adolescence, prevalence of sunburns remains unchanged, but the proportion who use sunscreen decreases [21]. Prior research indicates adolescent sun protective behaviors associated with fewer sunburns include staying inside and staying in the shade [20].

Existing research on presence of shade and adolescents has primarily examined school grounds [22]. Separately in New Zealand [23], Australia [24] and Germany [25], researchers found that the majority of school grounds or play equipment areas did not have shade and one recommendation was to plant trees with heavy foliage to increase shade. An examination of a built shade intervention on school grounds found that adolescents used the new shade provision in a sample of secondary schools in Australia [26]. In the U.S., it has been reported that lower socio-economic status schools have less playground shade than higher socio-economic status schools [27]. Past research has not examined how the availability of shade on school grounds may be associated with reported adolescent sunburns.

Previous studies have reported adolescent sunburns association with sun protective behaviors or have examined shade and potential UV exposure; yet, none so far have assessed these environmental factors (availability of shade cover and ambient UV) and individual behaviors with reported sunburns. In this study, we used measures of ambient UV as a proxy for adolescent UV exposure and we used measures of tree cover as a proxy for shade. The overall goal of this study was to quantify whether ambient UV and tree cover at adolescents’ home and school was associated with sunburns. Aim 1 was to examine adolescent sunburns, covariates and behaviors. Aim 2 was to assess the adolescent sunburns, covariates, behaviors and additionally the environmental variables of ambient UV and tree cover. A sub-aim of Aim 2 was sensitivity analyses to examine how ambient UV measures and tree cover buffer sizes were associated with sunburns. It is included online in Additional file 1. Aim 3 was to examine how the environmental variables varied across space with spatial analyses of model residuals.

## Methods

### Sample

We analyzed the publicly available U.S. National Cancer Institute’s (NCI) Family Life, Activity, Sun, Health, and Eating Study (FLASHE). FLASHE was a national web-based Ipsos Consumer Opinion Panel sample conducted in 2014 and fully enrolled 1945 parent–child dyads (a participation rate of 38.7%) from a total of 5027 dyads screened for eligibility [28]. Respondents provided information about demographics, such as age (aged 12–17 years), gender, race/ethnicity, employment; health behaviors; details of physical activity and sedentary time; and sunburns and sun protective behaviors [29].^1^ The sample for this study was adolescents who completed the physical activity survey, which included questions on sunburns and sun protective behaviors (n = 1661) [28]. Respondents missing responses to age (n = 37), gender (n = 5), race/ethnicity (n = 17), self-reported weight status (n = 7), sunburns (n = 6), any sun protective behaviors or tanning (n = 45), or typical physical activity, bike or walk to/from school or places (n = 20) were excluded. Those missing geocoded locations for home (n = 66) or school (n = 122) were also excluded. Finally, those missing environmental data (n = 2) or school neighborhood poverty data (n = 1) were excluded. After these exclusions, the analytic sample included 1333 adolescents. Participant geocoded home and school addresses were accessed in a restricted use and secure computing environment with Westat, Inc. to maintain confidentiality of participants’ information.

### Measures

#### Environmental measures

The environmental measures were ambient UV and tree cover. Ambient UV was measured by erythemal daily dose (EDD) and erythemally weighted irradiance (EDR). EDD represents the total amount of UV radiation that can cause sunburn over the course of a day [30]. EDR describes the amount of UV that can cause sunburn during midday; it is measured around noon when intensity is likely strongest [30]. These were daily measures at the county-level for the year 2014, from the Center for Disease Control and Prevention’s (CDC) Population-Weighted Ultraviolet Irradiance, 2004–2015 dataset [31]. The UV data were created by Emory University’s Environmental Remote Sensing Group using the Ozone Monitoring Instrument aboard the NASA Aura Spacecraft [30]. As adolescents likely have different UV exposure based on time of year, we subset the ambient UV by summer months (in the U.S.: June, July, and August) and non-summer months (in the U.S.: September to May), the latter roughly corresponding to the academic school year. We compared whether ambient UV during summer months or non-summer months was more strongly associated with sunburns, compared to complete year summary measures. Shade was approximated using the tree cover percentage analytical product, created by the U.S. Forest Service as part of the 2011 National Land Cover Database (NLCD) [32]. Percent tree cover is a measure of local tree density and was derived from 30 m Landsat imagery [32]. Percent tree cover was measured as continuous, 0%–100%. Tree cover was assessed for home and school geocoded locations separately. UV was summarized for home geocoded locations, as UV measures were nearly identical between home and school locations because of their geographic proximity.

Additional environmental measures were latitude, longitude, and school neighborhood poverty level. Latitude and longitude were from the geographic coordinates of the geocoded home point (decimal degrees; geographic coordinate system and datum: WGS 1984). School neighborhood poverty level was used to control for neighborhood socio-economic status around schools that may be associated with tree cover, based on a study in one urban area [27]. While it is unknown whether the school neighborhood poverty level and tree cover is a generalizable relationship [33], we include it to test whether it is significant with a national sample. School neighborhood poverty was defined as the percent of persons living below the federal poverty line from the American Community Survey 2010–2014. It was measured by the intersection of a 400 m buffer around the school geocoded location with Census tracts to produce a tract area weighted average of percentage poverty [34].

#### Outcome variable

The outcome variable was number of adolescent sunburns reported for the last 12 months; it was dichotomized to any or none, as is commonly done in sunburn research and to produce comparable results [13, 35]. The location of sunburns was not known.

### Data analysis

#### Geographic data processing

Environmental variables were assessed with Geographic Information System (GIS) buffers around home and school locations. Home and school locations were geocoded by Westat, Inc. using ArcGIS 10.4.1 with data from Esri's StreetMap Premium 2015, version 1 [34].^2^

### Statistical analysis

We reported unadjusted frequencies of any sunburns by covariates, behaviors, and environmental variables. We assessed unadjusted linear trends in the ordinal environmental variables using Cochran-Armitage two-sided trend tests. For covariate-adjusted models, only those covariates or behaviors that had significant bivariate associations with any sunburns were included. This was done to ensure we had reasonable power given our modest sample size and number of independent covariates. Age, sun protection behaviors, walk or bike to school or other locations, and home tree cover were not included in the adjusted analyses. Logistic regression^3^ was used to assess five models of association between the adjusted odds of any sunburns with: (1) covariates, behaviors, and no spatial variables; (2) covariates, behaviors, latitude and longitude; (3) covariates, behaviors, ambient UV, and longitude; (4) covariates, behaviors, tree cover, latitude and longitude; and (5) covariates, behaviors, ambient UV, tree cover, and longitude.^4^ We did not use a spatially explicit model form, but modeled potential spatial dependence in sunburns using latitude and longitude as continuous predictors.^5^*P* < 0.05 was used to determine statistical significance and SAS 9.4 (SAS Institute, Cary, NC, USA) was used for all statistical models and ArcGIS 10.7.1 (Esri, Redlands, CA, USA) was used for the geographic data operations and the spatial analysis of model residuals.

#### Assessment of model fit

Relative model fit was assessed with Akaike information criterion (AIC), where a lower relative score was equated with a better model fit and/or lower complexity [38]. Area under the curve (AUC) was used to compare model prediction quality. We assessed the models’ Receiver Operator Curve (ROC) estimates contrasted with the base model (model 1) ROC estimate using Chi-square tests.

#### Testing Spatial Dependence

Overall spatial dependence in the models’ error terms was tested with Global Moran’s *I* on the standardized deviance residuals at the home addresses [39, 40].^6^ We tested positive spatial autocorrelation of the standardized deviance residuals with Local Moran’s *I*, which indicates clusters of participants where the model over- (negative clusters) or under-predicted (positive clusters) sunburns. Due to confidentiality of the data, we did not present point-level maps of the Local Moran’s *I* results but reported the counts of participants in the negative and positive clusters for each model. We mapped the counts of participants within clusters as percentages of total participants for each Census division. This allowed us to identify overall and for regions of the country where there may be issues with model assumptions and fit.

## Results

### Sample characteristics

In the FLASHE sample, 44% of adolescents reported any sunburns in the past 12 months, with more females reporting sunburns (48.9%), compared to males (39.1%) (*p* = 0.0003) (Table 1). Those who reported any tanning bed use were more likely to report any sunburns (81.3%), compared to those who did not use tanning beds (43.0%) (*p* < 0.0001). A higher frequency of those who always or often used sunscreen reported more sunburns (50.1%) compared to those who sometimes, rarely, or never used sunscreen (41.1%) (*p* = 0.002). Lower categories of ambient UV were associated with sunburns (*p-trend* = 0.002). There was not a significant difference between home tree cover and reported sunburns (*p-trend* = 0.08), whereas lower categories of tree cover at school were associated with higher frequency of reported sunburns (*p-trend* = 0.008).

**Table 1:** Frequencies of covariates by any sunburns in the past 12 months, FLASHE 2014

	n	Any sunburns (%)		*p*
		Yes (n = 586)	No (n = 747)	
Overall	1333	44.0	56.0	
Male	673	39.1	60.9	0.0003^e^
Female	660	48.9	51.1	
Race/ethnicity^a^				
Hispanic	139	33.1	66.9	< 0.0001^e^
NH Black	214	10.3	89.7	
NH White	859	56.7	43.3	
NH Other	121	25.6	74.4	
Physical activity level in past 7 days				
None	132	34.1	65.9	0.04^e^
Sometimes/often	761	45.9	54.1	
Quite/very often	440	43.6	56.4	
Self-rated weight				
Very/a little under	145	46.9	53.1	0.009^e^
Just right	820	40.7	59.3	
Little/very over	368	50.0	50.0	
Any tanning bed use				
No	1301	43.0	57.0	< 0.0001^e^
Yes	32	81.3	18.8	
Sunscreen use				
Never/rarely/sometimes	908	41.1	58.9	0.002^e^
Often/always	425	50.1	49.9	
Sun protection (sleeves covering shoulders, hat, or seek shade)				
Never/rarely/sometimes	375	48.0	52.0	0.06^e^
Often/always	958	42.4	57.6	
Intentional sun exposure				
Never/rarely/sometimes	1131	40.3	59.7	< 0.0001^e^
Often/always	202	64.4	35.6	
Walk/bike to/from school^b^				
Any	281	44.1	55.9	0.95^e^
None	1052	43.9	56.1	
Home tree cover				
Low	444	46.2	53.8	0.08^f^
Medium	445	45.4	54.6	
High	444	40.3	59.7	
School tree cover				
Low	444	47.1	52.9	0.008^f^
Medium	445	46.5	53.5	
High	444	38.3	61.7	
UV^c^				
Low	444	49.1	50.9	0.002^f^
Medium	445	44.5	55.5	
High	444	38.5	61.5	
School poverty^d^				
Low	457	45.5	54.5	0.008^f^
Medium	419	50.1	49.9	
High	457	36.8	63.2	

*NH* non-Hispanic

^a^Other race is American Indian or Alaskan Native, Asian, or multiple races

^b^For adolescents not in school, this is walk/bike to/from a place, such as a job or friend’s house

^c^UV is the average daily EDR (mW m-2) for the academic year (September to May) for the county of participant residence

^d^School poverty was defined as the percent of persons living below the federal poverty line from the American Community Survey 2010–2014. It was measured by the intersection of a 400 m buffer around the school geocoded location with Census tracts to produce an area weighted average

^e^p-values are from Chi-square tests

^f^p-values are from Cochran-Armitage two-sided trend tests

Table 2 shows the average and standard deviations of sample age and home address latitude and longitude by any adolescent reported sunburns. Age was not significantly associated with sunburns (*p* = 0.33). On average, there was about one degree of latitude difference between adolescents who reported any sunburns (38.7 degrees (SD = 4.6)) compared to those who reported no sunburns (37.6 degrees (SD = 4.8)) (*p* < 0.0001). Those who reported sunburns lived about 122 km [42] further north than those who reported no sunburns, on average across the U.S. Results of the sensitivity analysis are available online as Additional File 1: Sensitivity Analysis.

**Table 2:** Unadjusted means and standard deviations of continuous variables by any sunburns

	Any sunburns		*p*
	Yes (n = 586)	No (n = 747)	
Age (years)	14.5 (1.6)	14.4 (1.6)	0.35
Latitude (decimal degrees north)	38.7 (4.6)	37.6 (4.8)	< 0.0001
Longitude (decimal degrees west)	− 90.4 (14.2)	− 89.3 (14.0)	0.14

p-values are from ANOVA *F*-tests

### Adjusted analyses

Table 3 presents the adjusted odds ratios of any reported sunburns for all covariates and the environmental variables of interest for the five models. To address Aim 1, model 1 included no spatial variables, only covariates and behaviors. The adjusted odds ratios (ORs) for sunburns were strongest for those who were non-Hispanic Black or African American, 0.09 (0.06–0.15) (*p* < 0.0001), those with any tanning bed use, 5.46 (2.03–14.64) (*p* = 0.0008) and those with any intentional sun exposure 1.78 (1.26–2.52) (*p* = 0.001).

**Table 3:** Adjusted odds ratios (ORs) of any sunburns for covariates and environmental variables of interest

	Model 1: No spatial variables		Model 2: Lat. and long.		Model 3: UV and long.		Model 4: Tree cover, lat. and long.		Model 5: UV, tree cover and long.	
	Adjusted ORs of any sunburns	*p*	Adjusted ORs of any sunburns	*p*	Adjusted ORs of any sunburns	*p*	Adjusted ORs of any sunburns	*p*	Adjusted ORs of any sunburns	*p*
Female (ref = male)	1.31 (1.03–1.68)	0.03	1.32 (1.03–1.69)	0.03	1.31 (1.03–1.68)	0.03	1.32 (1.03–1.69)	0.03	1.31 (1.02–1.68)	0.03
Race/ethnicity (ref = White, non-Hispanic)										
Hispanic	0.37 (0.25–0.55)	0.28	0.36 (0.24–0.54)	0.38	0.36 (0.24–0.54)	0.42	0.36 (0.24–0.54)	0.41	0.35 (0.24–0.53)	0.45
Black or African American, non-Hispanic	0.09 (0.06–0.15)	< 0.0001	0.10 (0.06–0.16)	< 0.0001	0.10 (0.06–0.16)	< 0.0001	0.10 (0.06–0.16)	< 0.0001	0.10 (0.06–0.16)	< 0.0001
Other, non-Hispanic	0.28 (0.18–0.43)	0.50	0.27 (0.17–0.42)	0.35	0.27 (0.17–0.42)	0.38	0.27 (0.17–0.41)	0.36	0.27 (0.17–0.42)	0.38
PA level in past 7 days (ref = none)										
Sometimes/often	1.36 (0.89–2.08)	0.22	1.32 (0.86–2.02)	0.23	1.32 (0.86–2.01)	0.24	1.30 (0.85–2.00)	0.26	1.30 (0.85–1.99)	0.26
Quite/very often	1.31 (0.84–2.06)	0.44	1.25 (0.79–1.97)	0.59	1.25 (0.80–1.97)	0.58	1.24 (0.79–1.95)	0.59	1.24 (0.79–1.96)	0.58
Self-rated weight (ref = very/a little under)										
Just right	0.76 (0.52–1.12)	0.005	0.74 (0.50–1.10)	0.004	0.75 (0.51–1.10)	0.004	0.73 (0.50–1.09)	0.004	0.74 (0.50–1.09)	0.004
Little/very over	1.22 (0.80–1.87)	0.03	1.18 (0.77–1.82)	0.04	1.19 (0.78–1.83)	0.04	1.17 (0.76–1.81)	0.04	1.18 (0.77–1.82)	0.04
Any tanning bed use (ref = no)	5.46 (2.03–14.64)	0.0008	5.23 (1.94–14.13)	0.001	5.36 (1.99–14.45)	0.0009	5.41 (2.00–14.71)	0.0009	5.45 (2.04–15.06)	0.0008
Sunscreen use (ref = never/rarely/sometimes)										
Often/always	1.00 (0.78–1.30)	0.99	1.00 (0.78–1.30)	0.98	1.01 (0.78–1.30)	0.95	1.00 (0.77–1.29)	0.99	1.00 (0.78–1.30)	0.98
Intentional sun exposure (ref = never/rarely/sometimes)										
Often/always	1.78 (1.26–2.52)	0.001	1.81 (1.28–2.56)	0.0008	1.82 (1.29–2.57)	0.0007	1.79 (1.26–2.53)	0.001	1.79 (1.27–2.54)	0.001
School neighborhood poverty (tertiles; linear trend)	0.97 (0.84–1.13)	0.71	0.97 (0.84–1.12)	0.67	0.96 (0.83–1.12)	0.61	0.97 (0.83–1.12)	0.65	0.96 (0.83–1.11)	0.60
Longitude	–	–	0.99 (0.98–1.00)	0.04	0.99 (0.98–1.00)	0.02	0.99 (0.98–1.00)	0.18	0.99 (0.98–1.00)	0.15
Latitude	–	–	1.03 (1.00–1.05)	0.06	–	–	1.02 (1.00–1.05)	0.11	–	–
Academic year; Average daily EDR (tertiles; linear trend)	–	–	–	–	0.89 (0.76–1.05)	0.17	–	–	0.92 (0.78–1.08)	0.29
School tree cover (tertiles; linear trend)	–	–	–	–	–	–	0.91 (0.78–1.07)	0.25	0.90 (0.76–1.07)	0.21

p-values are from Wald Chi-square tests

*Lat.* latitude, *long*. longitude

The subsequent models 2 through 5 addressed Aim 2 and assessed adolescent sunburns, covariates and behaviors and additionally ambient UV and tree cover. Model 2 added the spatial variables latitude and longitude to the covariates and behaviors in Model 1. Longitude was significant, with an adjusted OR of 0.99 (0.98–1.00) (*p* = 0.04), however, latitude was not significant, 1.03 (1.00–1.05) (*p* = 0.06). A one decimal degree increase in longitude, which corresponds to moving east, is associated with a 1% reduction in reported sunburns. Model 3 added the average ambient UV (EDR) during the academic year (September to May) categorized into tertiles and longitude. The adjusted linear association between ambient UV tertiles and any adolescent sunburns was not significant, with an OR of 0.89 (0.76–1.05) (*p* = 0.17). Model 4 added tree cover, latitude and longitude. The adjusted linear association between tree cover tertiles and any adolescent sunburns was not significant, with an OR of 0.91 (0.78–1.07) (*p* = 0.25). The final model, model 5, added ambient UV, tree cover and longitude; neither ambient UV nor tree cover were significantly associated with sunburns.

### Model fit

Table 4 presents assessments of model fit, predictive quality, and spatial analysis of the residuals. Model 2 has the lowest AIC, indicating that the tradeoff between model fit and/or lower complexity is the least, compared to the other models. The AUC estimates and Chi-square tests showed no significant difference in the predictive quality between the models 2–5 and model 1.

**Table 4:** Model fit, residual spatial dependence, and residual spatial autocorrelation comparisons

	AIC	Area Under Curve (AUC)	*p*^1^	Global Moran’s *I*^2^	*p*^3^	Count (%) of participants in significant, positive Local Moran’s *I* clusters		
						Positive^4^	Negative^5^	Total
Model 1	1603.897	0.736	Ref	0.0033	0.71	27 (2.0%)	58 (4.4%)	85 (6.4%)
Model 2	1600.689	0.742	0.14	-0.0028	0.85	25 (1.9%)	31 (2.3%)	56 (4.2%)
Model 3	1602.285	0.742	0.14	-0.0017	0.93	30 (2.3%)	42 (3.2%)	72 (5.4%)
Model 4	1601.381	0.744	0.09	-0.0033	0.81	28 (2.1%)	29 (2.2%)	57 (4.3%)
Model 5	1602.770	0.743	0.09	-0.0023	0.89	27 (2.0%)	34 (2.6%)	61 (4.6%)

^1^p-values are from Chi-square tests for differences in model Receiver Operator Curves (ROC) with Model 1 ROC

^2^Global Moran’s *I* test statistic based on the standardized deviance residuals at home addresses

^3^p-values are from z-scores based on a standard normal distribution of standardized deviance residuals

^4^Positive clusters represent significant (*p* < 0.05), positive clusters of home addresses with positive standardized deviance residuals (where the observed sunburn was higher than predicted)

^5^Negative clusters were significant (*p* < 0.05), positive clusters of home addresses with negative standardized deviance residuals (where the observed sunburn was lower than predicted)

Aim 3 was to examine how the environmental context variables varied across space with an examination of model residuals. This aim was assessed with the overall Global and Local Moran’s *I* statistics and with the results presented in the next section. The Global Moran’s *I* test for spatial dependence of the standardized deviance residuals at the home locations was not significant for all models, indicating no significant spatial dependence in the models. The counts and percentages of participants in significant (*p* < 0.05) Local Moran’s *I* positive and negative clusters indicated that model 2 had the fewest total participants in clusters (Table 4). Therefore, based on the AIC and Local Moran’s *I* statistics, model 2 appeared to be the preferred model for these data, relative to the other models.

### Spatial analyses

**Fig. 1:**
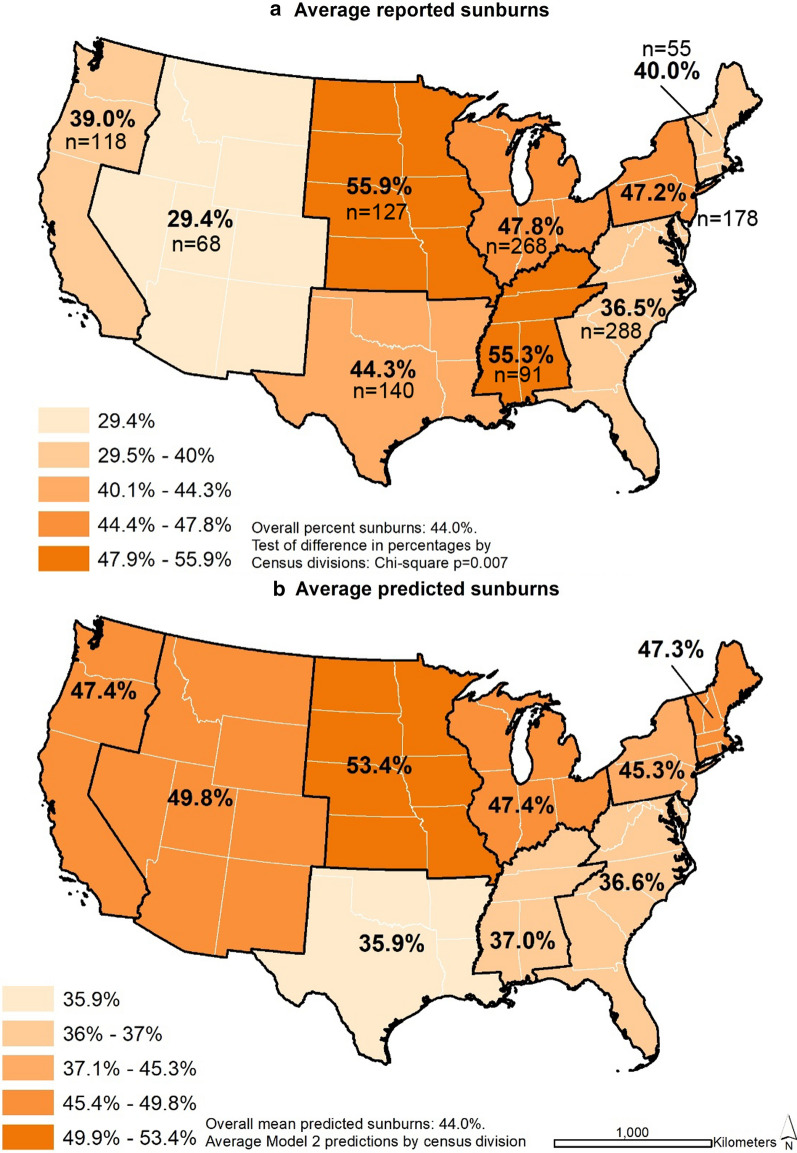
Unadjusted and number of participants (**a**) and adjusted (**b**) any adolescent reported sunburns in past 12 months by U.S. Census divisions, FLASHE 2014

Figure 1a illustrates the spatial distribution of unadjusted sunburns in the sample and there was a significant overall difference when stratified by Census divisions (*p* = 0.007). The Mountain division (n = 68), which consists of the states Arizona, New Mexico, Colorado, Wyoming, Utah, Montana, Idaho, and Nevada, had the lowest rate of sunburns at 29.4%. The West North Central division (n = 127), consisting of North Dakota, South Dakota, Nebraska, Kansas, Missouri, Iowa, and Minnesota, had the highest rate of sunburns at 55.9%. Figure 1b presents the average predicted probabilities of sunburn by Census divisions. The predicted probabilities were from model 2, which were adjusted for gender, race/ethnicity, typical weekly physical activity, weight status, sunscreen use, other sun protection behaviors, intentional sun exposure, tanning bed use, school neighborhood poverty, latitude and longitude. The predicted sunburns were similar to the observed sunburns in the East North Central (predicted: 47.4%; observed: 47.8%) and the South Atlantic (predicted: 36.6%; observed: 36.5%) divisions.

**Fig. 2:**
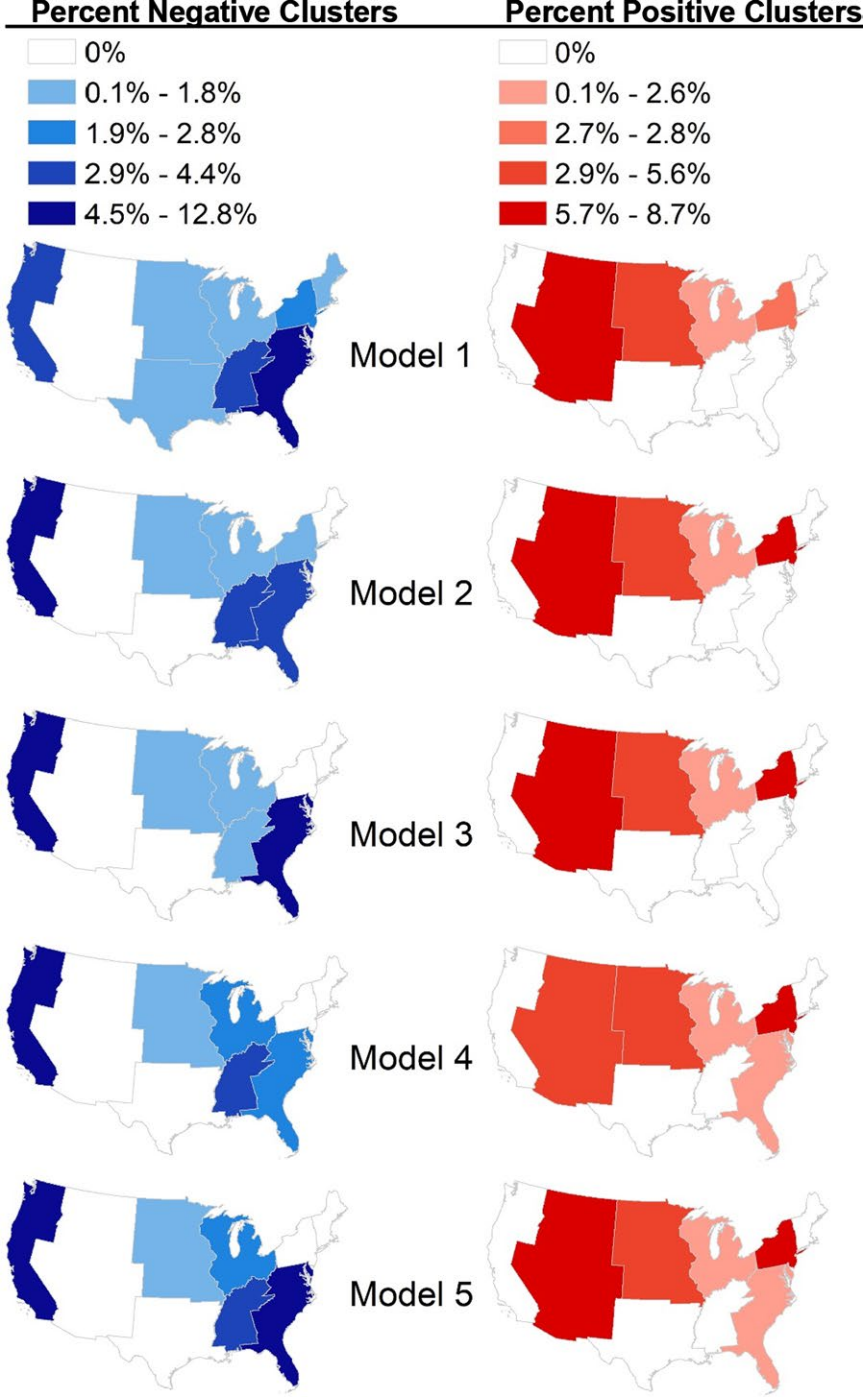
Percent of sample within Census division exhibiting spatial autocorrelation of residuals, based on significant, positive Local Moran’s *I* clusters

To address Aim 3 in further detail, Fig. 2 presents maps of the significant (*p* < 0.05) positive Local Moran’s *I* clusters based on the standardized deviance residuals for each model. It shows the percent of observations in each Census division that were in negative clusters, which represented where there was spatial clustering in the models over-predicting sunburns. The negative clusters were significant, positive spatial autocorrelation of the negative model residuals. Conversely, the positive clusters were significant, positive spatial autocorrelation of positive residuals, and showed where there was spatial clustering in where the models were under-predicting sunburns.

## Discussion

This paper did not find significant relationships between UV or tree cover and adolescent sunburns, when adjusted by sunburn-related covariates. Nevertheless, UV exposure does cause sunburn and better data are needed to evaluate the extent to which tree cover can reduce the risk of sunburn. Specifically, individual-level data on UV exposure, activity spaces, and school and neighborhood environments may provide an appropriate scale to assess the relationships. Our measure of UV exposure was approximated with ambient UV measures and our measure of shade was approximated with tree cover. We found that a significant negative bivariate association between ambient UV and sunburns, but the adjusted association was not significant. Previous research in U.S. adults from the 1999 Behavioral Risk Factor Surveillance System data found that the highest rates of sunburn in were in more northernly states, such as Wisconsin, Utah, Wyoming, Washington, DC, Indiana, Michigan, and Iowa [43]. Our results suggest that such associations may differ in different parts of the U.S. For Aim 1, we confirmed known associations between sunburn and gender, race/ethnicity, tanning bed use and intentional sun exposure. These items were more strongly associated with sunburns than the ambient UV summarized during the school year and tree cover on school grounds. For Aim 2, there was no association between adolescent sunburns, ambient UV and home or school tree cover, when adjusted by covariates. We did detect significant bivariate associations between any sunburns and lower average ambient UV during the months of the school year and higher tree cover around the school grounds. The sub-aim of Aim 2 (results in Additional file 1), the sensitivity analysis found that for ambient UV, there were four similar measures that discriminated sunburns. The tree cover estimates for the 200 m buffer distance around schools had the strongest association with sunburns. Conceptually, this distance was also the closest to approximating the area of school grounds.

Aim 3 of this study was to examine how the environmental variables varied across space, which was assessed with spatial analyses of the residuals. For regions with 0% in negative or positive clusters, this is what we would expect based on model assumptions: that the error terms of nearby participants were completely independent. It is important to note that these maps may differ from the overall average observed and predicted sunburns in Census divisions (Fig. 1) because the maps of residual clusters represent only a maximum of 12.8% of participants in negative clusters and 8.7% of participants in positive clusters in the divisions. What Fig. 2 instead highlights is that there were regions of the country where the model relationships may be unstable or where other explanatory factors that were not included in the models may be associated with sunburns. However, for the overall models, the extent of the correlation of the residuals were actually quite limited: concordant residuals range from 1.9% (n = 25) of total observations in model 2 within significant positive clusters to 4.4% (n = 58) of total observations in model 1 within significant negative clusters (Table 4). This indicates that a small percentage of observations may be subject to violation of the assumption of independence. Due to the small percentage of the total sample these represent, we conclude that this was a minimal amount of correlation and likely does not meaningfully violate the assumption of independence among the residuals or bias these models. Future research may explore parameters related to examining the spatial autocorrelation of model residuals for modeling sunburns, such as the geographic weighting function or size of the bandwidth.

The results from Aim 3 highlight the potential spatial variation in the associations between sunburns, covariates, behaviors and environments. Recent research reported adolescent sunscreen use, sun protective clothing use, and intentional sun exposure varied between three U.S. states [44]. For example, adolescents in Hawaii reported using sunscreen less frequently than those in California or Colorado [44]. Sun protective behaviors and sunburns also varied between urban and rural areas in Texas adults, with rural residents more likely to report using sunscreen with a higher sun protection factor (SPF), yet also report having had more sunburns during adolescence compared to urban residents [45]. We documented significant clustering of model residuals that may suggest some of the coefficient variance and significance tests may be biased. However, these results also suggest that there may be omitted explanatory variables that may explain regional clusters in sunburns, which would provide additional context beyond the spatial coordinates, ambient UV, and tree cover we included. Some explanatory variables may be urban or rural residence or proximity to a beach. This may be addressed with spatially explicit geographic data (e.g., geocoded locations or Global Positioning System (GPS) receivers to track individuals’ UV exposure) and detailed participant-reported contexts (such as at pool or beach) to more accurately model the relationship between sunburns, behaviors, and environments.

The characterization of shade for each school grounds was a challenge. First, this required an accurate definition of school grounds area in which to assess shade. To do so would have required obtaining parcel data for each school and these data are held by counties for tax collection purposes. This data acquisition work would have required contacting hundreds of counties and combining their data to identify the school grounds for the schools in this sample. Second, identifying natural and built shade on school grounds would have required using Google Earth [27] or other aerial imagery, in addition with ground truthing, to accurately identify the variety of trees and built shade structures on school grounds. Unfortunately, we did not have the resources to address either of these issues, which is why we used estimates of tree cover summarized with buffers. The accuracy of the nationwide estimates of tree cover from the NLCD varied. The 2011 percent tree cover was predicted using a random forests models, which used aerial imagery, 2001 NLCD tree cover predictions, elevation, and Landsat data as predictors. The aerial imagery training data were from 5 areas in Georgia, Michigan, Kansas, Oregon and Utah, where researchers used photo-interpretation to classify each of the 105 points, per sample location, as either “tree canopy” or “no tree canopy” using the National Agriculture Imagery Program (NAIP) imagery [32]. The pseudo-R^2^ for models in these regions ranged from 0.53 to 0.90; however, the standard errors of predicted tree cover were higher for urban areas [32]. Additionally, the NLCD estimates of tree cover we used will likely differ from estimates made in situ or from 0.5 m or 5 m resolution imagery [46]. This likely led to some misclassification of the tree cover percentage at home and school locations, which was one reason why we classified the percentages into tertiles. The direction of this potential misclassification of tree cover is likely to be non-differential and the bias in the relative associations with sunburns would be towards the result of a null association; that is, not a statistically significant association [47]. However, further conditions must hold for the assumption of the bias to be towards the null association, such as the condition that the misclassification probabilities are exactly non-differential; exposure misclassification errors are assumed to be independent of errors in other variables in the analysis; and, for the associations to be towards the null also assume absence of interactions with other sources of systematic error, such as selection bias and confounding [48]. Finally, the temporal match between the 2011 NLCD tree cover product and the 2014 FLASHE survey, which asked about sunburns the previous 12 months, which covered part of 2013 was not a perfect match. The next more recent tree cover product is the 2016 NLCD. So, there was not a perfect match temporally from either data product, however we feel the 2011 product was a reasonable approximation of the tree cover in 2013 and 2014. Future research may seek to model tree cover at yearly intervals to allow a better temporal match with health surveys.

Environmental and policy interventions to promote reduction of UV exposure among adolescents require consideration of context. In general, the Community Preventive Services Taskforce has found sufficient evidence to recommend environmental and policy supports to minimize UV exposure for the contexts of childcare settings [49] and primary and middle schools [50]. At childcare and these two school settings, environmental and policy interventions include increasing the availability of sun-protective items (e.g., sunscreen or protective clothing), employing policies (e.g., protective clothing guidelines or restrictions on outdoor activities during peak UV hours) and increasing sun-protective features of the physical environment (e.g., shade) [49, 50]. The provision of shade interventions to reduce UV exposure among adolescents at schools is possible through built structures, such as portable shade tents or permanent solid roof or fabric installations, or natural features, such as trees [6–8, 22, 51, 52]. In addition to school grounds, school-associated activities, such as athletic sporting events, are recommended to provide shade through environmental supports to reduce adolescent UV exposure, as other sun protective behaviors among participants and spectators are minimal [53]. Finally, more evidence is required to evaluate the effectiveness of the above interventions for high school and college settings [54].

## Strengths and limitations

One strength of this study was a national sample, although not nationally representative. The national sample allowed variation in environmental contexts, socio-demographics, and behaviors. For example, adolescent sun protective behaviors vary between some U.S. states [44], so using a national sample allowed for more generalizable results than a study located in a specific city, state or region. Another strength was the geocoded home and school locations, which allowed estimates of local tree cover. Few other studies have assessed sunburns and tree cover with this level of detail, with most assessing either sunburns and behaviors [18, 19, 21, 44] or tree cover and potential UV exposure [6, 23, 25–27]. While our measure of sunburns was broad (over previous 12 months), we leveraged ambient UV, important sun protective behaviors, and other covariates to explore the role of tree cover with sunburns.

This paper had three main limitations. First, it lacked information on skin sensitivity to the sun, which has a more nuanced association with sunburns than race/ethnicity alone [13]. This limitation may have resulted in the odds ratio estimates for race/ethnicity to be stronger than if skin sensitivity was included as well. Second, the measure of sunburns was broad: any sunburns in the past 12 months. These sunburns could have occurred at any time during those months or at any location, such as on vacation or at the local pool, and therefore measures of ambient UV and tree cover around home and schools may not have captured the environments of these other sunburn contexts. This finding represents an example of the uncertain geographic context problem, where there may be a spatial mismatch between measurements of the behavior and putative environmental influences [55]. This spatial mismatch may influence the results by either overstating the role of the environment associated with behaviors or by not capturing the role of environment associated with behaviors. Third, the use of tree cover as the measure of shade did not include built environment shade, which may have resulted in an under-estimate of the shade environments of schools. More comprehensive data, such as combining participant worn GPS with UV dosimeters and participant reported outcomes (i.e., sunburns, sun protective behaviors) may allow better modeling of the relationships between ambient UV, tree cover, and adolescent sunburns to guide future interventions. Finally, while we explored different representations of UV and tree cover, such as continuous or ordinal tertiles, we did not find a qualitative difference in associations with sunburns. Future research may explore other means of representing UV and tree cover for explaining variations in sunburns.

## Conclusions

The relationships between ambient UV, tree cover, and adolescent sunburns, as an important life stage to study a key risk factor for melanoma, is an area that needs further research in the U.S. and internationally. Currently, there is an absence of nationwide studies to examine adolescent sunburns and the environment. This analysis is one of the first studies to assess ambient UV, tree cover, and adolescent sunburns using a national sample. This study did not find significant associations between adolescent sunburns and environmental measures. These results, which did not support our hypotheses and the spatial analysis of the residuals, suggest two recommendations for future research. First, that more comprehensive data on the environmental and activity contexts of adolescent sunburns are necessary. And second, more accurate measures of shade, that includes both built and natural shade are needed. These improved data may allow a better accounting of the costs and benefits of environmental shade interventions to inform policies to reduce adolescent UV exposure and sunburns for skin cancer prevention.

## Supplementary Information

**Additional file 1.** Sensitivity Analysis. Description, figure, and table of sensitivity analyses performed.

**Additional file 2.** Model Comparison. Table comparing logistic and negative binomial sunburn models.

## Data Availability

The study analyzed the publicly available (https://cancercontrol.cancer.gov/brp/hbrb/flashe.html) and restricted access FLASHE datasets. Data use agreements, to protect the confidentiality of participants’ data, were signed by all authors.

## Footnotes

1 Coding of covariates was as follows: Weight status was recoded from five categories to three: very underweight or a little underweight; just right; and, very overweight or a little overweight. The sun protective behavior measure was a composite of three different questions on frequency during summer on a warm sunny day. The three behaviors included: 1) wearing a shirt with sleeves covering shoulders; 2) wearing a hat; and 3) seeking shade or using an umbrella. The sun protective behavior measure was coded as yes if the respondent answered always or often to any of the questions and was coded as no otherwise. Sunscreen was not combined with these behaviors, because previous research has found a positive association with sunburns [13]. Sunscreen was recoded as yes if response was often or always and no if response was sometimes; rarely; or, never. Intentional sun exposure (spending time in the sun in order to get a tan), was recoded as yes if the response was often or always and recoded as no if response was sometimes; rarely; or, never. Any tanning booth or bed use in the past 12 months was recoded as yes if response was ≥ 1 time or no (0 times). Physical activity was measured with a question on typical weekly physical activity level and recoded to three categories none; sometimes/often; and, quite/very often. A separate question on frequency of days walking or biking to school or other locations in the past week was recoded to any, ≥ 1 time, or none.

2 Geocoded home locations resulted in primarily point locations (73%) or street addresses (18%) [34]. Some participants chose not to report their home locations (8%). The rest were geocoded to an intersection (0.3%), street name (0.7%), or ZIP code (0.1%). Geocoded school locations resulted in mainly street intersections (78%), as the survey asked about the nearest cross streets of the adolescents’ school [34]. The rest were street name (0.2%), ZIP code (3.8%), or not reported or refused (18%). We excluded those with missing or not reported geocodes.

3 We also assessed another model form, the negative binomial generalized linear model, in which the sunburns dependent variable was modeled as a count variable. This yielded qualitatively similar results to the logistic regression models (Supplemental File 2- Model Comparison).

4 Latitude was not included in models with ambient UV, because when tested for multicollinearity in a linear regression model, the variance inflation factor (VIF) was greater than 5. This is one recommended cut-off point where inclusion of correlated covariates can become problematic due to poor coefficient estimates [36]. We did not examine an adjusted model of tree cover around home with sunburns because the bivariate association was not significant. Longitude was included as a measure of continuous spatial variation in the adjusted models, even though the bivariate association with sunburns was not significant.

5 We fit a Geographically Weighted General Linear Model (GWGLM) with these data [37], but did not include the results. There were two main reasons why this model specification was unsuitable. First, the number of neighbors that was required for the best fitting GWGLM, based on minimizing the AICc, was a model where each local regression required 200 of the nearest neighbors. That is, 15% of the total observations were required to estimate the local regression models for each observation. We believed that this was a rather large amount of the sample and did not represent a feasible neighborhood for a local regression model. Second, the spatial neighborhood that these 200 neighbors spanned was many states for some observations. Again, the spatially sparse sample resulted in large geographic neighborhoods for the local regression models, and the addition of such spatially distant information is not likely to be informative for the local regression models.

6 Spatial dependence of the residuals was important to assess, as this may lead to biased variance estimates, tests of coefficient significance and model fit statistics [40]. Global assessment of spatial dependence of residuals may overlook local areas of spatial autocorrelation among residuals. If there are significant local clustering of residuals, that is an indicator of model instability over space, that is the relationships between variables change over space [41].
