# Supplementary material for: Associations between ultraviolet radiation, tree cover and adolescent sunburns
Source: Int J Health Geogr. 2020 Dec 14;19:59. doi: 10.1186/s12942-020-00253-x (PMC7734787; doi:10.1186/s12942-020-00253-x)
Supplement: Supplementary file 1 — Additional file 1. Sensitivity Analysis. Description, figure, and table of sensitivity analyses performed. [file 12942_2020_253_MOESM1_ESM.docx]

|  | Model 1: No spatial variables | | | | Model 2: Lat. and long. | | | | | |
| --- | --- | --- | --- | --- | --- | --- | --- | --- | --- | --- |
|  | Logistic | | Negative Binomial | | Logistic | | | Negative Binomial | | |
|  | Adjusted ORs  of any sunburns | *p* | Coefficient Estimates | *p* | Adjusted ORs  of any sunburns | *p* | Coefficient Estimates | | *p* |  |
| Female (ref=male) | 1.31  (1.03-1.68) | 0.03 | 0.14  (-0.02-0.30) | 0.08 | 1.32  (1.03-1.69) | 0.03 | 0.14  (-0.02-0.30) | | 0.08 |  |
| Race/ethnicity (ref=White, non-Hispanic) |  |  |  |  |  |  |  | |  |  |
| Hispanic | 0.37  (0.25-0.55) | 0.28 | -0.47  (-0.73- -0.21) | 0.0003 | 0.36  (0.24-0.54) | 0.38 | -0.50  (-0.77- -0.24) | | 0.0002 |  |
| Black or African  American, non-Hispanic | 0.09  (0.06-0.15) | <0.0001 | -1.55  (-1.89- -1.21) | <0.0001 | 0.10  (0.06-0.16) | <0.0001 | -1.49  (-1.83- -1.15) | | <0.0001 |  |
| Other, non-Hispanic | 0.28  (0.18-0.43) | 0.50 | -0.86  (-1.19- -0.54) | <0.0001 | 0.27  (0.17-0.42) | 0.35 | -0.89  (-1.21- -0.57) | | <0.0001 |  |
| PA level in past 7 days (ref=none) |  |  |  |  |  |  |  | |  |  |
| Sometimes/often | 1.36  (0.89-2.08) | 0.22 | 0.21  (-0.07-0.49) | 0.15 | 1.32  (0.86-2.02) | 0.23 | 0.19  (-0.09-0.47) | | 0.18 |  |
| Quite/very often | 1.31  (0.84-2.06) | 0.44 | 0.24  (-0.06-0.53) | 0.12 | 1.25  (0.79-1.97) | 0.59 | 0.21  (-0.09-0.50) | | 0.17 |  |
| Self-rated weight (ref=very/a little under) |  |  |  |  |  |  |  | |  |  |
| Just right | 0.76  (0.52-1.12) | 0.005 | -0.08  (-0.32-0.16) | 0.50 | 0.74  (0.50-1.10) | 0.004 | -0.09  (-0.33-0.15) | | 0.46 |  |
| Little/very over | 1.22  (0.80-1.87) | 0.03 | 0.15  (-0.11-0.41) | 0.26 | 1.18  (0.77-1.82) | 0.04 | 0.13  (-0.13-0.40) | | 0.31 |  |
| Any tanning bed use (ref=no) | 5.46  (2.03-14.64) | 0.0008 | 0.82  (0.44-1.20) | <0.0001 | 5.23  (1.94-14.13) | 0.001 | 0.80  (0.42-1.17) | | <0.0001 |  |
| Sunscreen use (ref= never/rarely/sometimes) |  |  |  |  |  |  |  | |  |  |
| Often/always | 1.00  (0.78-1.30) | 0.99 | 0.09  (-0.07-0.25) | 0.26 | 1.00  (0.78-1.30) | 0.98 | 0.09  (-0.07-0.25) | | 0.25 |  |
| Intentional sun exposure (ref=never/rarely/sometimes) |  |  |  |  |  |  |  | |  |  |
| Often/always | 1.78  (1.26-2.52) | 0.001 | 0.42  (0.24-0.61) | <0.0001 | 1.81  (1.28-2.56) | 0.0008 | 0.44  (0.25-0.63) | | <0.0001 |  |
| School neighborhood poverty | 0.97  (0.84-1.13) | 0.71 | -0.03  (-0.12-0.07) | 0.58 | 0.97  (0.84-1.12) | 0.67 | -0.03  (-0.12-0.06) | |  |  |
| Longitude | - | - |  |  | 0.99  (0.98-1.00) | 0.04 | -0.01  (-0.01- -0.0001) | | 0.02 |  |
| Latitude | - | - |  |  | 1.03  (1.00-1.05) | 0.06 | 0.01  (-0.002-0.03) | | 0.08 |  |
| Academic year; Average daily EDR (tertiles; linear trend) | - | - | - | - | - | - |  | |  |  |
| School tree cover (tertiles; linear trend) | - | - | - | - | - | - |  | |  |  |

|  | Model 3: UV and long. | | | | Model 4: Tree cover, lat. and long. | | | | | |
| --- | --- | --- | --- | --- | --- | --- | --- | --- | --- | --- |
|  | Logistic | | Negative Binomial | | Logistic | | | Negative Binomial | | |
|  | Adjusted ORs  of any sunburns | *p* | Coefficient Estimates | *p* | Adjusted ORs  of any sunburns | *p* | Coefficient Estimates | | *p* |  |
| Female (ref=male) | 1.31  (1.03-1.68) | 0.03 | 0.14  (-0.02-0.30) | 0.08 | 1.32  (1.03-1.69) | 0.03 | 0.14  (-0.02-0.30) | | 0.08 |  |
| Race/ethnicity (ref=White, non-Hispanic) |  |  |  |  |  |  |  | |  |  |
| Hispanic | 0.36  (0.24-0.54) | 0.42 | -0.51  (-0.77- -0.24) | 0.0002 | 0.36  (0.24-0.54) | 0.41 | -0.50  (-0.77- -0.24) | | 0.0002 |  |
| Black or African  American, non-Hispanic | 0.10  (0.06-0.16) | <0.0001 | -1.50  (-1.83- -1.16) | <0.0001 | 0.10  (0.06-0.16) | <0.0001 | -1.49  (-1.83- -1.15) | | <0.0001 |  |
| Other, non-Hispanic | 0.27  (0.17-0.42) | 0.38 | -0.89  (-1.21- -0.57) | <0.0001 | 0.27  (0.17-0.41) | 0.36 | -0.89  (-1.21- -0.57) | | <0.0001 |  |
| PA level in past 7 days (ref=none) |  |  |  |  |  |  |  | |  |  |
| Sometimes/often | 1.32  (0.86-2.01) | 0.24 | 0.19  (-0.09-0.47) | 0.19 | 1.30  (0.85-2.00) | 0.26 | 0.18  (-0.10-0.47) | | 0.20 |  |
| Quite/very often | 1.25  (0.80-1.97) | 0.58 | 0.20  (-0.09-0.50) | 0.18 | 1.24  (0.79-1.95) | 0.59 | 0.20  (-0.10-0.50) | | 0.18 |  |
| Self-rated weight (ref=very/a little under) |  |  |  |  |  |  |  | |  |  |
| Just right | 0.75  (0.51-1.10) | 0.004 | -0.09  (-0.33-0.15) | 0.46 | 0.73  (0.50-1.09) | 0.004 | -0.10  (-0.34-0.15) | | 0.44 |  |
| Little/very over | 1.19  (0.78-1.83) | 0.04 | 0.14  (-0.13-0.40) | 0.31 | 1.17  (0.76-1.81) | 0.04 | 0.13  (-0.13-0.40) | | 0.32 |  |
| Any tanning bed use (ref=no) | 5.36  (1.99-14.45) | 0.0009 | 0.80  (0.43-1.18) | <0.0001 | 5.41  (2.00-14.71) | 0.0009 | 0.80  (0.43-1.18) | | <0.0001 |  |
| Sunscreen use (ref= never/rarely/sometimes) |  |  |  |  |  |  |  | |  |  |
| Often/always | 1.01  (0.78-1.30) | 0.95 | 0.09  (-0.06-0.25) | 0.25 | 1.00  (0.77-1.29) | 0.99 | 0.09  (-0.07-0.24) | | 0.27 |  |
| Intentional sun exposure (ref=never/rarely/sometimes) |  |  |  |  |  |  |  | |  |  |
| Often/always | 1.82  (1.29-2.57) | 0.0007 | 0.44  (0.25-0.63) | <0.0001 | 1.79  (1.26-2.53) | 0.001 | 0.43  (0.25-0.62) | | <0.0001 |  |
| School neighborhood poverty | 0.96  (0.83-1.12) | 0.61 | -0.03  (-0.12-0.06) | 0.47 | 0.97  (0.83-1.12) | 0.65 | -0.03  (-0.12-0.06) | | 0.50 |  |
| Longitude | 0.99  (0.98-1.00) | 0.02 | -0.007  (-0.01- -0.002) | 0.01 | 0.99  (0.98-1.00) | 0.18 | -0.005  (-0.01- 0.001) | | 0.10 |  |
| Latitude | - | - | - | - | 1.02  (1.00-1.05) | 0.11 | 0.01  (-0.004-0.03) | | 0.12 |  |
| Academic year; Average daily EDR (tertiles; linear trend) | 0.89  (0.76-1.05) | 0.17 | -0.07  (-0.17-0.03) | 0.15 | - | - | - | | - |  |
| School tree cover (tertiles; linear trend) | - | - | - | - | 0.91  (0.78-1.07) | 0.25 | -0.04  (-0.14-0.06) | | 0.41 |  |

|  | Model 5: UV, tree cover and long. | | | |
| --- | --- | --- | --- | --- |
|  | Logistic | | Negative Binomial | |
|  | Adjusted ORs  of any sunburns | *p* | Coefficient Estimates | *p* |
| Female (ref=male) | 1.31  (1.02-1.68) | 0.03 | 0.14  (-0.02-0.30) | 0.08 |
| Race/ethnicity (ref=White, non-Hispanic) |  |  |  |  |
| Hispanic | 0.35  (0.24-0.53) | 0.45 | -0.51  (-0.77- -0.25) | 0.0002 |
| Black or African  American, non-Hispanic | 0.10  (0.06-0.16) | <0.0001 | -1.49  (-1.83- -1.15) | <0.0001 |
| Other, non-Hispanic | 0.27  (0.17-0.42) | 0.38 | -0.89  (-1.21- -0.57) | <0.0001 |
| PA level in past 7 days (ref=none) |  |  |  |  |
| Sometimes/often | 1.30  (0.85-1.99) | 0.26 | 0.18  (-0.10-0.46) | 0.20 |
| Quite/very often | 1.24  (0.79-1.96) | 0.58 | 0.20  (-0.10-0.50) | 0.19 |
| Self-rated weight (ref=very/a little under) |  |  |  |  |
| Just right | 0.74  (0.50-1.09) | 0.004 | -0.09  (-0.34-0.15) | 0.45 |
| Little/very over | 1.18  (0.77-1.82) | 0.04 | 0.13  (-0.13-0.40) | 0.32 |
| Any tanning bed use (ref=no) | 5.45  (2.04-15.06) | 0.0008 | 0.81  (0.43-1.19) | <0.0001 |
| Sunscreen use (ref= never/rarely/sometimes) |  |  |  |  |
| Often/always | 1.00  (0.78-1.30) | 0.98 | 0.09  (-0.07-0.25) | 0.26 |
| Intentional sun exposure (ref=never/rarely/sometimes) |  |  |  |  |
| Often/always | 1.79  (1.27-2.54) | 0.001 | 0.43  (0.25-0.62) | <0.0001 |
| School neighborhood poverty | 0.96  (0.83-1.11) | 0.60 | -0.03  (-0.13-0.06) | 0.47 |
| Longitude | 0.99  (0.98-1.00) | 0.15 | -0.006  (-0.01- 0.0004) | 0.07 |
| Latitude | - | - | - | - |
| Academic year; Average daily EDR (tertiles; linear trend) | 0.92  (0.78-1.08) | 0.29 | -0.06  (-0.16-0.04) | 0.22 |
| School tree cover (tertiles; linear trend) | 0.90  (0.76-1.07) | 0.21 | -0.04  (-0.15-0.06) | 0.40 |
