# Supplementary material for: Associations between ultraviolet radiation, tree cover and adolescent sunburns
Source: Int J Health Geogr. 2020 Dec 14;19:59. doi: 10.1186/s12942-020-00253-x (PMC7734787; doi:10.1186/s12942-020-00253-x)
Supplement: Supplementary file 2 — Additional file 2. Model Comparison. Table comparing logistic and negative binomial sunburn models. [file 12942_2020_253_MOESM2_ESM.docx]

Additional file 1: Sensitivity Analysis

Associations between ultraviolet radiation, tree cover and adolescent sunburns

Calvin P. Tribby, Anne K. Julian, April Y. Oh, Frank M. Perna, and David Berrigan

*Description*. In the literature, there is no consensus for which ambient UV measures, summary types, or time period is most appropriate for assessing adolescent sunburns. Also, there is no consensus on what the most appropriate buffer size is to measure and summarize tree cover for associations with adolescent sunburns. For these two reasons, we performed sensitivity analyses for measures of UV and buffer distances for tree cover. For ambient UV, we assessed the association between the two different measures (EDD and EDR) and sunburns, with three summary types (average, maximum, and cumulative) by three time periods (summer, academic year, and full year). We selected the ambient UV measure associated with sunburns based on the highest coefficient of determination from bivariate associations with sunburns. For tree cover, we assessed the association between sunburns and the average percent tree cover around home and school geocodes within three circular buffer distances: 200 m, 400 m, and 1000 m (Figure 1). Finally, ambient UV and tree cover were assessed as continuous measures or as ordinal tertiles.

Figure 1. Example of the sensitivity analysis for the percentage tree cover buffer distances. This was a randomly chosen school with the street intersection geocoded and actual school grounds.


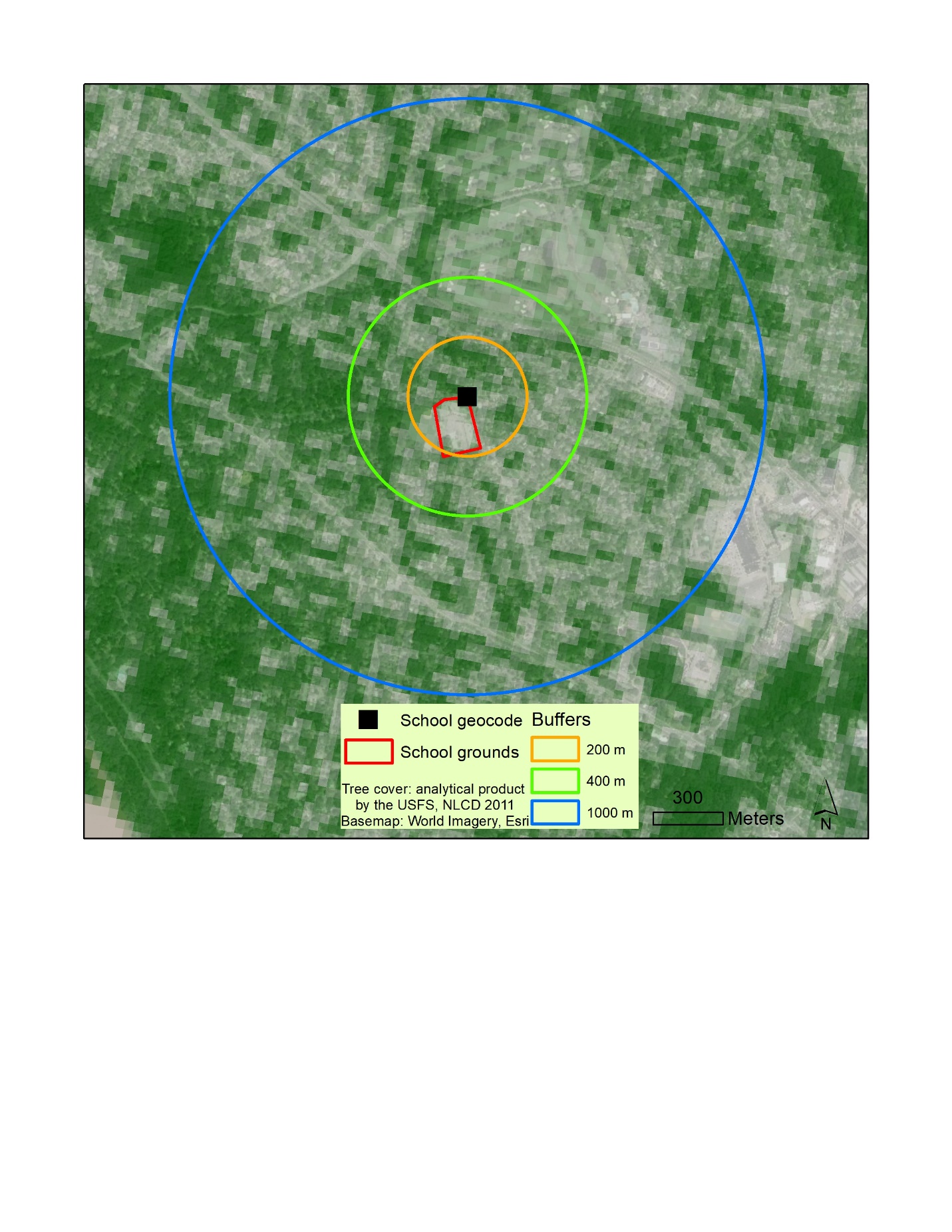


*Results*. Sub-aim of Aim 2 was a sensitivity analysis of ambient UV and tree cover measures. The buffer distance of 200 m was selected as most relevant for assessing tree cover and sunburns, as 400 m (home: p-trend=0.16; school: p-trend=0.07) and 1000 m (home: p-trend=0.05; school: p-trend=0.09) were not as strongly associated with sunburns and were not significant. Finally, 400 m and 1000 m distances encompassed much more area than typical school grounds in the US (see Figure 1).

Table 3 presents the sensitivity analysis of the two measures of ambient UV (EDD and EDR) and the summary types (average, maximum and cumulative). The summary time period of the academic year (September through May) for the ambient UV measures of EDD and EDR had the same coefficient of determination (0.009) for average and cumulative daily summaries. The average and standard deviations for the daily EDD for the academic year was 1917.3 J m-2 (546.4) for those who reported any sunburns and 2024.9 J m-2 (561.4) for those who reported no sunburns (p=0.0005). The average and standard deviations for the daily EDR for the academic year was 96.7 mW m-2 (28.9) for those who reported any sunburns and 102.5 mW m-2 (29.7) for those who reported no sunburns (p=0.0004). We selected the average daily EDR for the academic year as our ambient UV measure that we converted to tertiles; however, it performed similarly to the other three measures mentioned.

Table 3. Sensitivity analysis of sunburns with UV measures (EDD and EDR) by summary type (average, maximum and cumulative) and time of year (total; summer (June, July, August); and, academic (September through May))

|  | Any sunburns | | *p* | R^2^ |
| --- | --- | --- | --- | --- |
|  | Yes (n=586) | No (n=747) |  |  |
| Average daily EDD (J m^-2^) ^a^ |  |  |  |  |
| Year total | 2514.8 (568.6) | 2614.6 (576.1) | 0.002 | 0.007 |
| Summer | 4307.5 (670.8) | 4383.5 (662.6) | 0.04 | 0.003 |
| Academic | 1917.3 (546.4) | 2024.9 (561.4) | 0.0005 | 0.009 |
| Maximum daily EDD (J m^-2^) |  |  |  |  |
| Year total | 5861.7 (564.6) | 5881.8 (546.5) | 0.52 | 0.0003 |
| Summer | 5826.7 (590.8) | 5855.9 (564.5) | 0.36 | 0.0006 |
| Academic | 5476.8 (484.9) | 5502.9 (475.4) | 0.33 | 0.0007 |
| Cumulative daily EDD (J m^-2^) |  |  |  |  |
| Year total | 729098.0 (179868.5) | 760140.5 (185882.1) | 0.002 | 0.007 |
| Summer | 332600.0 (64579.8) | 340965.4  (65626.9) | 0.02 | 0.004 |
| Academic | 396498.1 (117934.2) | 419175.1 (123656.9) | 0.0007 | 0.009 |
| Average daily EDR (mW m^-2^) ^b^ |  |  |  |  |
| Year total | 120.9 (29.6) | 126.4 (30.2) | 0.001 | 0.008 |
| Summer | 193.7 (33.1) | 198.2 (32.9) | 0.01 | 0.005 |
| Academic | 96.7 (28.9) | 102.5 (29.7) | 0.0004 | 0.009 |
| Maximum daily EDR (mW m^-2^) |  |  |  |  |
| Year total | 254.9 (28.8) | 257.3 (28.5) | 0.13 | 0.002 |
| Summer | 252.9 (30.3) | 255.8 (29.6) | 0.08 | 0.002 |
| Academic | 241.2 (24.9) | 244.0 (25.0) | 0.05 | 0.003 |
| Cumulative daily EDR (mW m^-2^) |  |  |  |  |
| Year total | 34798.7 (9227.65) | 36474.1 (9565.2) | 0.001 | 0.008 |
| Summer | 14954.5 (3152.2) | 15421.8 (3208.5) | 0.008 | 0.005 |
| Academic | 19844.2 (6172.3) | 21052.3 (6484.5) | 0.0006 | 0.009 |

^a^ Erythemal daily dose (EDD) represents the total amount of UV radiation that can cause sunburn over the course of a day.

^b^ Erythemally weighted irradiance (EDR) describes the amount of UV that can cause sunburn during midday. It is measured around noon when intensity is likely strongest.

*P*-values are from ANOVA tests

*Discussion*. We found differences in the bivariate associations between adolescent sunburns and ambient UV. Across summary types (average, maximum and cumulative) and ambient UV measurements (EDD and EDR), the time period that best predicted sunburns was the academic year (September through May), compared to the full year or summer only periods. Also, the average and cumulative summary types for EDD and EDR had similar discrimination between sunburns, which is similar to previous research examining average and cumulative ambient UV and melanoma outcomes [43,44].
